# Supplementary material for: Estimation of genomic prediction accuracy from reference populations with varying degrees of relationship
Source: PLoS One. 2017 Dec 21;12(12):e0189775. doi: 10.1371/journal.pone.0189775 (PMC5739427; doi:10.1371/journal.pone.0189775)
Supplement: S1 Fig — Although a substantial proportion includes close relationships with an effective population size of 50, the mean and variance of the genomic relationships is -0.001and 0.0072, respectively, which agrees with the expected value from the theory (0 and 0.0077). This indicates that Eq (5) is valid with any random sample even having close relationship. (DOCX) [file pone.0189775.s001.docx]

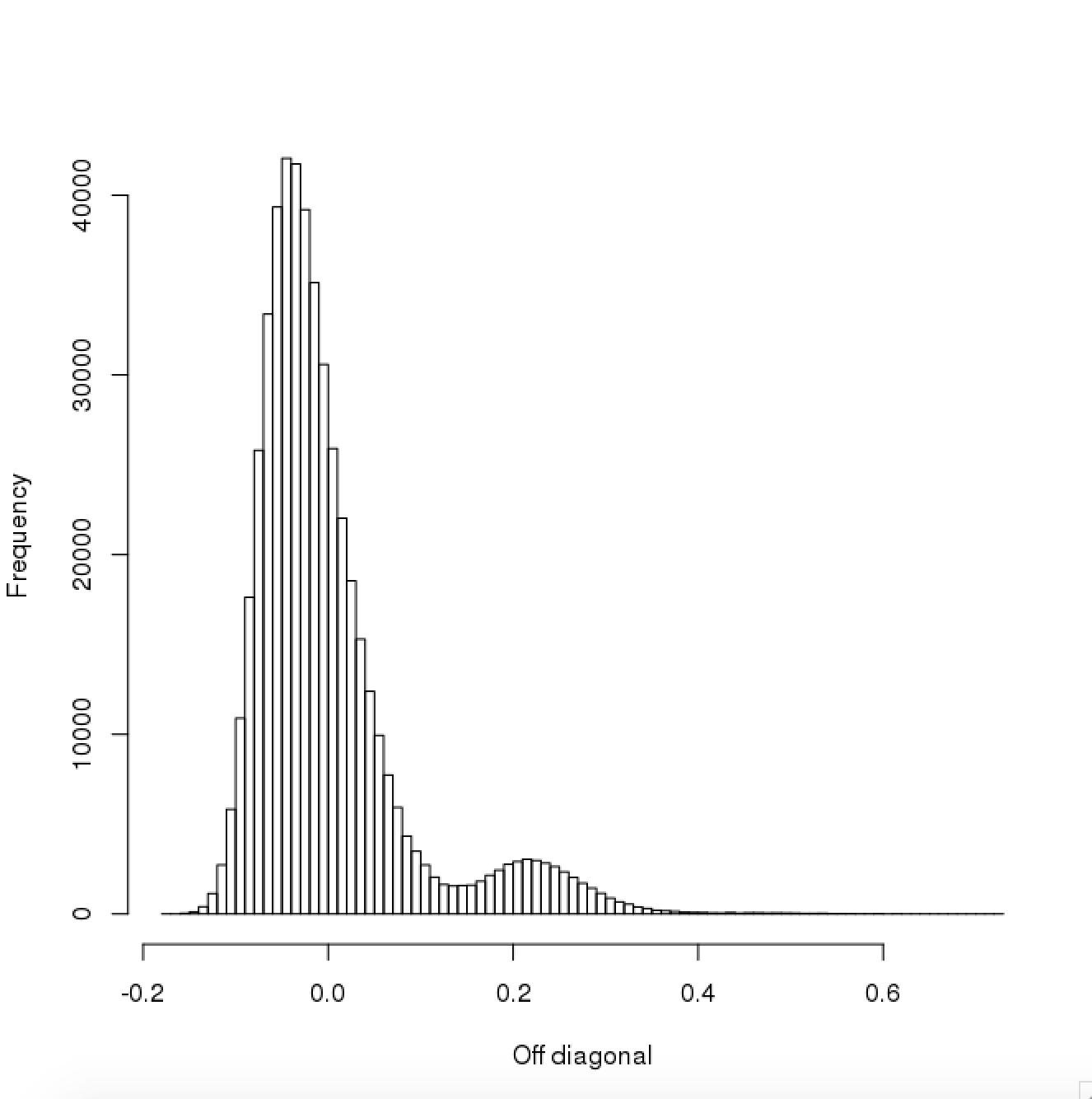


S1 Fig. The distribution of off-diagonal of the genomic relationships matrix among 1000 individuals when effective population size is 50.

Although a substantial proportion includes close relationships with an effective population size of 50, the mean and variance of the genomic relationships is -0.001and 0.0072, respectively, which agrees with the expected value from the theory (0 and 0.0077). This indicates that Eq. (5) is valid with any random sample even having close relationship.
