# Supplementary material for: Estimation of genomic prediction accuracy from reference populations with varying degrees of relationship
Source: PLoS One. 2017 Dec 21;12(12):e0189775. doi: 10.1371/journal.pone.0189775 (PMC5739427; doi:10.1371/journal.pone.0189775)
Supplement: S2 Fig — With an effective population size of 1000 (having hardly close relationships as expected), the mean and variance of the genomic relationships is -0.001 and 0.0004, respectively, which agrees with the expected value from the theory (0 and 0.0004). It is noted that whether using a high or low effective population size, the mean and variance of genomic relationships is generally matched with the expectation, supporting that any random samples from the population can used for Eq (5). (DOCX) [file pone.0189775.s002.docx]

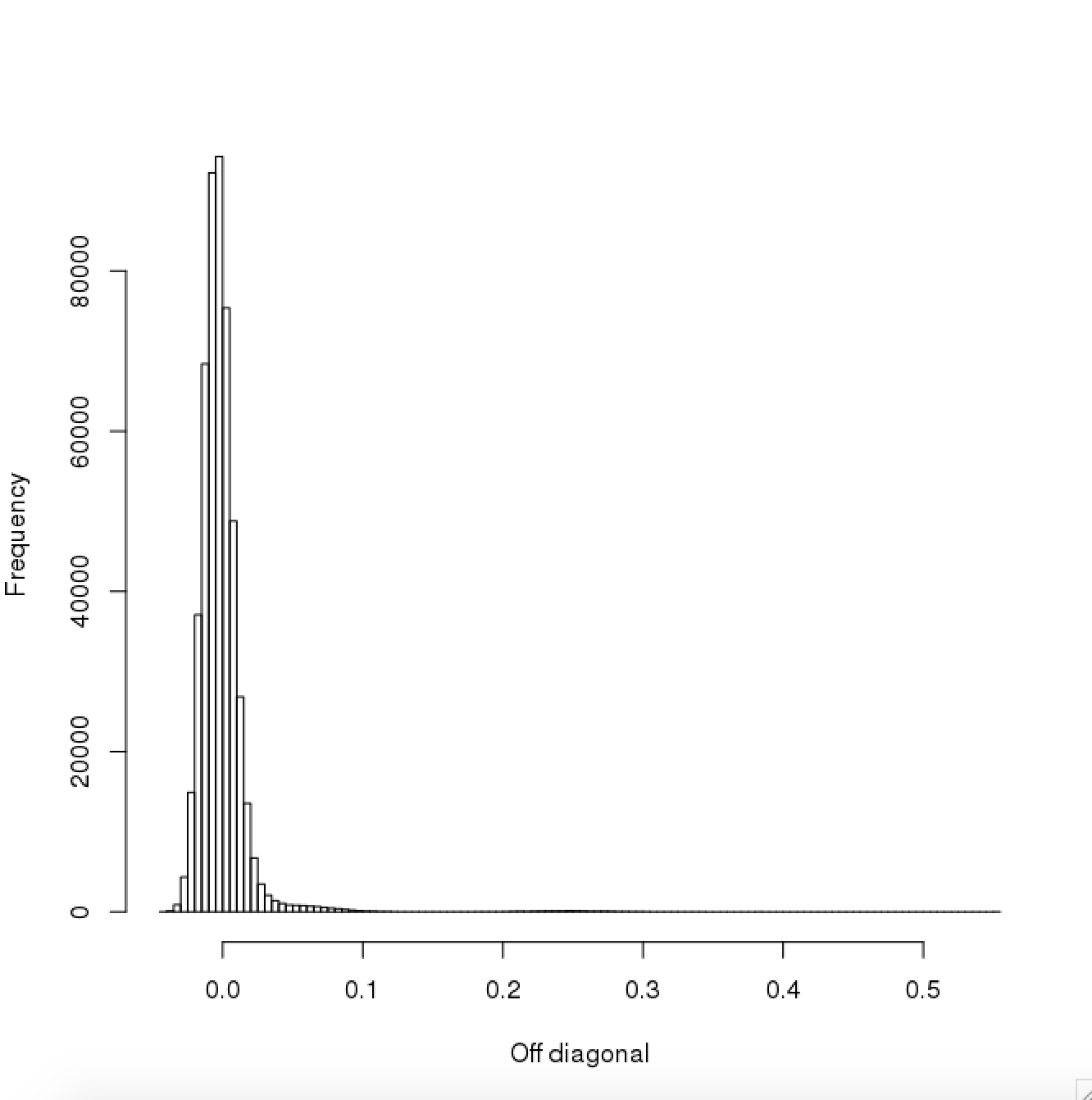


S2 Fig. The distribution of off-diagonal of the genomic relationships matrix among 1000 individuals when effective population size is 1000.

With an effective population size of 1000 (having hardly close relationships as expected), the mean and variance of the genomic relationships is -0.001 and 0.0004, respectively, which agrees with the expected value from the theory (0 and 0.0004). It is noted that whether using a high or low effective population size, the mean and variance of genomic relationships is generally matched with the expectation, supporting that any random samples from the population can used for Eq. (5).
