# Supplementary material for: Estimation of genomic prediction accuracy from reference populations with varying degrees of relationship
Source: PLoS One. 2017 Dec 21;12(12):e0189775. doi: 10.1371/journal.pone.0189775 (PMC5739427; doi:10.1371/journal.pone.0189775)
Supplement: S2 Table — Given the family structure, we estimated variance of relationships, which can be used to obtain Me from Eq (5). Assuming that the number of chromosomes is 30 chromosomes and the length of each chromosome is 1 Morgan (total 30 Morgan), the effective population size was estimated from Eq (2). (DOCX) [file pone.0189775.s004.docx]

S2 Table. Approximated value for effective population size (*N_e_*) for structured population of half sib families (kinship coefficient of 0.25)

| Number of families | Number of members within family | | | | | | | | | | | |
| --- | --- | --- | --- | --- | --- | --- | --- | --- | --- | --- | --- | --- |
|  | ***2*** | ***5*** | ***10*** | ***20*** | ***30*** | ***40*** | ***50*** | ***60*** | ***70*** | ***80*** | ***90*** | ***100*** |
| ***2*** | 23 | 24 | 24 | 24 | 24 | 24 | 24 | 24 | 24 | 24 | 24 | 24 |
| ***5*** | 62 | 45 | 41 | 40 | 39 | 39 | 39 | 39 | 39 | 39 | 39 | 39 |
| ***10*** | 128 | 84 | 77 | 73 | 72 | 72 | 71 | 71 | 71 | 71 | 71 | 71 |
| ***20*** | 264 | 167 | 150 | 142 | 140 | 139 | 138 | 138 | 138 | 137 | 137 | 137 |
| ***30*** | 402 | 252 | 225 | 213 | 210 | 208 | 207 | 206 | 206 | 206 | 205 | 205 |
| ***40*** | 542 | 338 | 301 | 285 | 280 | 278 | 277 | 276 | 275 | 274 | 274 | 274 |
| ***50*** | 682 | 425 | 377 | 358 | 351 | 348 | 347 | 346 | 345 | 344 | 344 | 343 |
| ***60*** | 824 | 512 | 455 | 431 | 423 | 420 | 417 | 416 | 415 | 414 | 414 | 413 |
| ***70*** | 967 | 599 | 532 | 504 | 495 | 491 | 488 | 487 | 486 | 485 | 484 | 484 |
| ***80*** | 1110 | 688 | 610 | 578 | 568 | 563 | 560 | 558 | 557 | 556 | 555 | 554 |
| ***90*** | 1255 | 776 | 689 | 652 | 641 | 635 | 632 | 630 | 628 | 627 | 626 | 625 |
| ***100*** | 1399 | 865 | 767 | 726 | 714 | 707 | 704 | 701 | 700 | 698 | 697 | 697 |

Given the family structure, we estimated variance of relationships, which can be used to obtain *M_e_* from Eq. (5). Assuming that the number of chromosomes is 30 chromosomes and the length of each chromosome is 1 Morgan (total 30 Morgan), the effective population size was estimated from Eq. (2).
